# New Appliances and Things Medical

**Published:** 1900-11-24

**Authors:** 


### NEW APPLIANCES AND THINGS MEDICAL.

[We shall be glad to receive, at our Office, 28 & 29 Southampton Street, Strand, London, W.C., from the manufacturers, specimens of all new preparations and appliances which may be brought out from time to time.]

### HÆMOGALLOL AND STYPTICIN.

(E. Merck, 16 Jewry, London, E.C.)

Hæmogallol is an organic iron preparation obtained by a process invented by Professor Kobert, which comprises the reduction of hæmoglobin by such bodies as zinc dust and pyrogallol. The resulting body contains a large percentage of iron, and fulfils the conditions which are now demanded of iron preparations if they are directly to assist in the formation of hæmoglobin in the body. These conditions may be generally stated as follows:—The iron must be absorbed from the alimentary tract; it must remain in the system for an appreciable period—for instance a few days; and part of it must be excreted by the kidneys in combination with uric acid. An iron preparation fulfilling these conditions may be assumed to have entered into close combination with the iron containing proteids of the body, and hence in a large measure to have corrected the conditions which are responsible for the anæmic state. Three or four 5-grain tabloids of hæmogallic may be regarded as one of the best medicinal treatments of anæmia. Stypticin is an oxidation product of narcotin, an alkaloid of opium; in its therapeutic and chemical properties it resembles hydrastinine, and is useful in the same conditions for which the latter drug is prescribed; that is to say, for menorrhagia and other uterine disorders. It is a styptic with anodyne properties. It should be given in 1-grain doses three or four times a day.

### THE ALLENBURYS SERIES OF INFANT FOODS.

(Allen and Hanburys, Limited, Plough Court, Lombard Street, E.C., and Bethnal Green, London, E.)

Although we are well acquainted with the Allenburys method of infant feeding, we have recently examined more carefully into the details on which depend the success or failure of the practical application. We ourselves have occasionally observed failures, and we have heard of others, and in each case we have come to the conclusion that the directions supplied with the foods have not been implicitly followed. But at the same time we cannot altogether absolve the manufacturers from blame. Their directions are not sufficiently precise for so delicate a matter as infant feeding. For instance, a child one day old should not be given the same quantity as a child one week old; if this information is to be gathered from the directions, it is by implication and not directly. The system is well conceived, and if carried out exactly and scientifically should certainly, and can, as we are able personally to testify, give most excellent results. The objects which are fulfilled by the method are manifold. In the first place the basis of the food is milk in a specially prepared and modified form—when made according to the directions it approximates to the chemical constitution of maternal milk. It can be digested far more easily than ordinary forms of diluted and modified milk, and is especially suitable for delicate and premature infants. Secondly, the three varieties of food are appropriately modified to the digestive powers of the infant during the successive periods of its life. The milk food No. 1 is intended for the first three months of life, and is prepared for use by mixing with water only. It is the only food of the kind with which we are acquainted which contains a sufficiently high percentage of fat for the requirements of infants at this age. If the amount of sugar in the food as prepared for use could be reduced from 10 per cent. to 6 per cent., without interfering with the percentage of the fat, the food would be practically perfect, if supplemented, as the manufacturers advise, or rather insist, with some antiscorbutic element, such as meat or grape juices. The milk food No. 2 is similar to food No. 1, but contains in addition maltose, dextrine, soluble phosphates, and albuminoids. When mixed according to the directions it again approximates to the constitution of maternal milk, and affords an easy stepping-stone to the third stage of the series. This food No. 3 is a partially digested farinaceous food, which should be added to diluted milk. This latter food, though qualitatively excellent, should be given in measured and exact quantities. Owing to the ease with which carbohydrates can be given in excess during this stage (i.e., six months to a year), we are again of opinion that the directions are not sufficiently precise. Taken as a whole, the Allenbury method is an excellent one: it provides variety in well-regulated gradation in accordance with the infant power of digestion. The possibility of infection by means of contaminated milk is impossible—at any rate during the first six months—and scurvy rickets should not ensue if occasionally fresh food as directed is made a part of the régime.

